# Optimization of underground open intermediary space comfort in TOD complexes: A case study of Chongqing, China

**DOI:** 10.3389/fpubh.2023.1108750

**Published:** 2023-02-24

**Authors:** Dong Lili, He Yufeng, Chen Xiang, Cheng Na, Liu Tao

**Affiliations:** ^1^College of Architecture and Urban Planning, Chongqing Jiaotong University, Chongqing, China; ^2^Faculty of Architecture and Urban Planning, Chongqing University, Chongqing, China; ^3^Key Laboratory of the Ministry of Education of Mountainous City and Towns Construction and New Technology, Chongqing University, Chongqing, China; ^4^Chongqing City Integrated Transportation Hub (Group) Co., Ltd., Chongqing, China

**Keywords:** TOD complexes, underground open intermediary space, physical environment, comfort optimization, numerical simulation

## Abstract

Rapid urbanization drives social development, but at the same time brings sustainable development Rapid urbanization drives social development, but at the same time brings sustainable development advantages of expanding underground space and relieving urban traffic congestion. High quality TOD complexes with natural elements in the intermediary space have been considered as one of the important means to address sustainable urban development. Nevertheless, intermediary spaces in TOD complexes face various challenges, such as significant contradictory factors in their physical environment spaces. This study classifies the underground open intermediary space into four types according to the characteristics of TOD complexes. And for these four types'Cthe physical environment—generated by various influencing factors of planar geometric, three-dimensional geometric, and detailed construction elements—is simulated using a numerical simulation method based on a static Taguchi experiment. The results demonstrate that space shape is a primary influencing factor for luminous and thermal environments; the window-atrium ratio (W/A ratio) and hole-atrium ratio (H/A ratio) comprise contradictory factors between the luminous and thermal environments of these spaces; profile inclination angle and sunken plaza height are primary impact factors for the acoustic environment; and skylight type has minimal influence on the physical environment. On average, their luminous and acoustic environment comfort can be improved by 200%; whereas, their thermal environment comfort can be improved by 21% and the potential for optimizing it in their shallow space (underground space depth ≤ 10 m) is relatively low. Subsequently, the necessity of comfort optimization as the passive optimization design of underground open intermediary spaces' physical environment in TOD complexes in the future is discussed. Finally, the feasible path and prospect of how to improve the livability and comfort of the spatial physical environment of TOD complexes are discussed and prospected.

## 1. Introduction

Global urbanization has brought about a nearly 20% increase in the global urban population in the twenty-first century, and is expected to account for more than 70% of the global population by the mid-twenty-first century (1, 2). The accelerating urbanization process has induced a number of problems such as construction land shortage, sharp decreases in ecological space, increased energy consumption, and a decline in human settlement quality, posing a continuous threat to urban sustainability. Hence, human settlements are facing unprecedented opportunities and challenges. To promote smart growth and intensive and green development of cities, the United Nations adopted the New Urban Agenda to define the future orientation of urban development, or specifically, to encourage sustainable urban planning, promote transit-oriented development (TOD) urban construction, enhance urban resilience, and improve the quality of urban public space (3). The New Urban Agenda states that expanding underground space and developing TOD complexes can alleviate regional traffic congestion, maintain a balanced ratio of workers to residents, and ensure the flexibility, inclusiveness, and sustainability of urban habitats (4), thus facilitating the development of 15-min cities. Existing studies on TOD complexes have mainly focused on planning-related fields (5) and their impact on the built environment, or more specifically, their impact on the surrounding communities (6, 7) and historical buildings (8).

As the concept of TOD complexes is increasingly perfected, multi-point interconnected, vertically stereoscopic, multi-functional, and downward-extending underground intermediary space has emerged in the TOD complexes of developed countries. Despite numerous advantages, underground intermediary space in TOD complexes is inherently closed, arousing a sense of discomfort (e.g., depression and irritation) among indoor occupants (9) and other negative consequences to people's health (10, 11). A luminous environment is among the essential environmental factors that maintain human health, and an absence of natural light induces symptoms such as biorhythmic disturbance and reduced concentration, especially among the staff in such spaces. Even though artificial auxiliary lighting is widely used in the underground intermediary space of TOD complexes, the luminous environment cannot meet both visual and non-visual health needs. The thermal environment is among the major environmental factors affecting the human body's perception of temperature (12), and an uncontrolled thermal environment will significantly reduce people's comfort (13), and can result in manic depression and boredom (14, 15). The acoustic environment is among the primary impact factors for the efficiency of information spread, work and learning, and human health. In most TOD complexes, the unreasonable design of underground intermediary space leads to severe non-compliance with some indices such as reverberation time and average noise (16). Prolonged stay in a space with a poor physical environment can induce various symptoms (e.g., loss of accurate time judgment, deterioration of vision and memory, reduced work efficiency, and even hallucinations) (17). To improve the use efficiency and human settlement quality of underground intermediary space in TOD complexes, introducing natural elements and maintaining a harmonious physical environment are key (18).

Fortunately, underground open intermediary space in TOD complexes offers a good solution to the above problems. First, natural light can be better utilized through passive techniques, thus improving its luminous environment (19). This can be done through reasonable design (e.g., increasing the lighting depth and optimizing the geometric parameters) to create a comfortable luminous environment. Regarding thermal environment optimization, thermal discomfort in summer can be alleviated by reducing the skylight area and daylighting hours. However, it is usually difficult to achieve the desired effect by optimizing the luminous or thermal environment, respectively. The optimization of the luminous environment parameters may lead to an uncomfortable thermal environment; for example, an excessive increase in the skylight coverage ratio may cause overly high indoor temperatures in summer (20). Therefore, the design of related parameters must consider the indoor luminous-thermal balance (21). Recent studies have proved that the use of passive techniques is a feasible way to maintain a comfort balance between luminous and thermal environments (22), and that low-cost technologies can be compatible with indoor environmental comfort (23) (Table 1). For example, certain devices (e.g., double-layered skylights) are considered an effective way to alleviate thermal discomfort in underground intermediary spaces (24); despite some additional costs, such devices are still cost-effective optimization solutions due to their implicit value to people's physical and mental health. Moreover, further cost saving can be achieved by adjusting the design parameters (e.g., space shape and skylight type). The adjustment of luminous and thermal parameters may also effectively optimize acoustic comfort by changing the indoor reverberation time and sound pressure level by altering the space shape and volume (25).

**Table 1:** A review of research methods, optimization goals, and parameter targets of previously studied open intermediary spaces.

**References**	**Building type**	**Type of open intermediary space**	**Method**	**Optimization object**	**Target parameter**
Guo et al. (27)	Commercial building	Sunken plaza	Numerical simulation	Thermal environment	Orientation and geometric parameter
Ghasemi et al. (28)	Office building	Atrium space	Numerical simulation	Light environment	Spatial geometric ratio
Zhao et al. (25)	Commercial building	Atrium space	Numerical simulation	Acoustic environment	Skylight type and length-width ratio
Brothánek et al. (29)	Public building	Atrium space	Numerical simulation Field measurement	Acoustic environment	Number of users
Yang et al. (14)	Not specified	Atrium space	Numerical simulation	Thermal environment	Window location
Ghasemi et al. (28)	Office building	Atrium space	Numerical simulation	Light environment	Spatial geometric ratio
Fan et al. (30)	Public building	Atrium space	Numerical simulation	Light environment	Photovoltaic material
Dai and Jiang (31)	Public building	Atrium space	Numerical simulation	Luminous and thermal environments	Skylight coverage ratio

In the recent decade, there have been few studies of the overall physical environment of underground open intermediary space (18); the target parameters of such studies mainly focused on planar geometric elements (e.g., skylight coverage ratio and space shape), solid geometric elements (e.g., spatial height and profile inclination angle), and detailed construction elements (e.g., skylight type and photovoltaic materials). Methodologically, previous studies almost unanimously adopted numerical simulations to optimize the indoor environment, but did not conduct any general surveys of architectural space or detailed space classifications, thus reducing the universality of conclusions.

To fill this gap, this study classified the underground intermediary space of different TOD complexes. Regarding functional orientation and spatial opening form, the underground open intermediary spaces of 28 TOD complexes in Chongqing were classified into four types, including underground atrium intermediary space in a TOD urban complex (TUAI), underground atrium intermediary space in a TOD commercial complex (TCAI), underground atrium intermediary space in a TOD hub complex (THAI), and sunken plaza intermediary space in a TOD commercial complex (TCSI). Based on the data size of spaces measured, a standard model of the four types of spaces was established and input into Ecotect for the analysis of the indoor physical environment, and the target parameters for optimization were summarized based on the feedback from field research. Finally, the potential for optimizing the four types of space was analyzed through a comparison between the best and worst combination of impact factors.

## 2. Methodology

### 2.1. Developing a protocol

**Figure 1:**
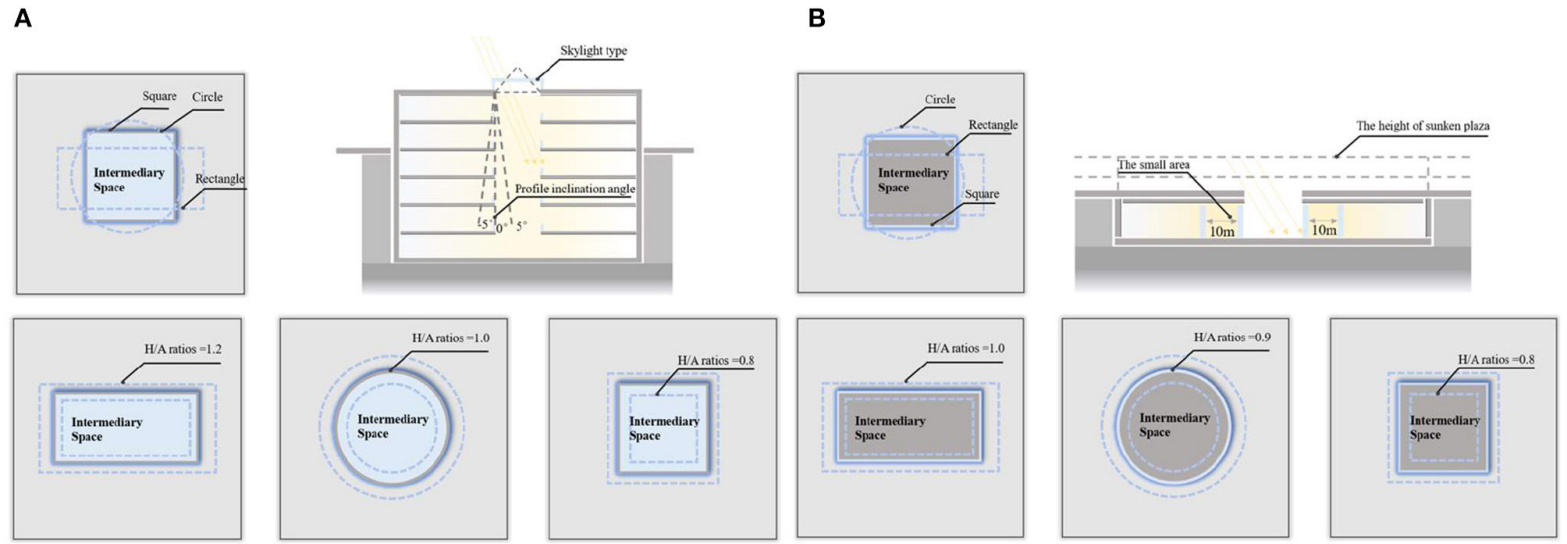
Protocol describing the logic for changing the combination of factors in the simulation. Image source: self-drawn by the author. **(A)** Underground atrium type intermediary space. **(B)** Sunken plaza type intermediary space.

We developed a separate protocol for simulating the physical environment of two types of open intermediary space to assist in constructing an overall simulation framework and analyze their physical environment (26). As illustrated in Figure 1, this protocol describes the logic for changing the combinations of the factors involved in the simulation process, and clearly expresses the range of impact factors. A more reasonable value range of related parameters can be determined by considering diverse factors (e.g., space shape, profile inclination angle, W/A ratio, and skylight type) comprehensively rather than separately, thereby providing higher accuracy than conventional methods.

### 2.2. Combination of conditions

#### 2.2.1. Experimental design of TUAI, TCAI, and THAI

To ensure that the influence of the above impact factors on the physical environment of buildings was not disturbed by other factors, we only changed the combination of impact factors and the variation range of level factors. TUAI, TCAI, and THAI all fall under underground atrium intermediate space, thus their impact factors share the same variation range with level factors. The data herein were cited from architectural survey data (Table 2). The status table of impact factors and their level factors was generated after the constant conditions, impact factors, and their level factors were determined (Table 3). A total of 81 experiments were conducted according to the full factorial design method. To reduce the experimental cost and improve the experimental efficiency, the static Taguchi method was adopted, and the target conditions were determined by the orthogonal table (Table 3). The static Taguchi method is usually used in multi-objective optimization studies, and is widely recognized in the product design field (32–34). Experimental schemes describe the logic and range of value variation of impact factors, meaning different TOD complexes share the same experimental scheme. Figure 1A pertains to the TUAI, while TCAI and THAI are not separately discussed in detail.

**Table 2:** The statistics of space size information form field research.

**Location**	**Number of aboveground floors**	**Number of underground floors**	**Height of space (m)**	**Space area (m^2^)**	**Skylight area (m^2^)**	**Space shape**	**Length-width ratio**
Shapingba station	5	7	66	600	600	Square	1:1
Shapingba station	5	0	30	400	400	Rectangle	1:2
Xiaoshizi station	1	1	17	650	300	Rectangle	1:3
Xiaoshizi station	3	2	26	1,000	1,000	Rectangle	1:2
Xiaoshizi station	3	2	26	120	120	Circle	1:1
Ranjiaba station	1	4	30	200	250	Square	1:1
Xuetangwan station	4	1	25	700	700	Rectangle	1:3
Xuetangwan station	4	1	25	1,000	1,000	Rectangle	1:4
Chongqing west station	1	2	18	700	700	Circle	1:1
Chongqing west station	1	2	18	700	700	Circle	1:1

**Table 3:** Impact factors, level factors, and orthogonal table of TUAI, TCAI, and THAI.

**Impact factors and level factors**				
	**Impact factors**	**Level factor 1**	**Level factor 2**	**Level factor 3**
A	Space shape	Rectangle	Circle	Square
B	Skylight type	Arched skylight	Flat skylight	Rectangular skylight
C	W/A ratio	0.8	1.0	1.2
D	Profile inclination angle	−5°	0°	5°
	**Orthogonal table L9(3** ^4^ **)**			
**Conditions**	**Space shape**	**Skylight type**	**W/A ratio**	**Profile inclination angle**
1	Rectangle	Arched	0.8	−5°
2	Rectangle	Flat	1.0	0°
3	Rectangle	Rectangular	1.2	5°
4	Circle	Arched	1.0	5°
5	Circle	Flat	1.2	−5°
6	Circle	Rectangular	0.8	0°
7	Square	Arched	1.2	0°
8	Square	Flat	0.8	5°
9	Square	Rectangular	1.0	−5°

#### 2.2.2. Experimental design of TCSI

The spatial form of TCSI is different from that of TUAI, TCAI, and THAI, so the impact factors for its physical environment are different as well. TCSI has a higher degree of exposure, so the impact factors for its physical environment are fewer than those for TUAI, TCAI, and THAI. As mentioned in Section 2.2.1, the space shape, H/A ratio, and sunken plaza height were surveyed on-site based on a literature review, and the level factors of impact factors were sourced from the field measurement data of the TOD complexes in Chongqing. The status table of impact factors and their level factors were generated after they were determined. Finally, conditions 1–9 were determined through the orthogonal table L9(3^3^) (Table 4).

**Table 4:** Impact factors, level factors, and orthogonal table of TCSI.

**Impact factors and level factors**				
	**Impact factors**	**Level factor 1**	**Level factor 2**	**Level factor 3**
A	Space shape	Rectangle	Circle	Square
B	H/A ratio	0.8	0.9	1.0
C	Sunken plaza height	5 m	6 m	7 m
	**Orthogonal table L9(3** ^3^ **)**			
**Conditions**		**Space shape**	**Skylight type**	**W/A ratio**
1		Rectangle	Arched	0.8
2		Rectangle	Flat	1.0
3		Rectangle	Rectangular	1.2
4		Circle	Arched	1.0
5		Circle	Flat	1.2
6		Circle	Rectangular	0.8
7		Square	Arched	1.2
8		Square	Flat	0.8
9		Square	Rectangular	1.0

### 2.3. Evaluation index of the physical environment

To reflect the quality of the physical environment, the simulation results of conditions were converted into response data for a direct evaluation of comfort. In this study, comfort evaluation included four parts, namely, luminous environment evaluation, thermal environment evaluation, acoustic environment evaluation, and physical environment evaluation.

1. Luminous environment evaluation. The subjective comfort and spaciousness of people in a high-illuminance space are always higher than those in a low-illuminance space (35). In this study, the luminous environment was evaluated in terms of average illuminance. For the luminous environment response data, S_1_ was used to indicate the mean value of the average illuminance of all floors (for single-floor space, average illuminance was used as the response data). The larger the S_1_ value, the better the luminous environment.
2. Thermal environment evaluation. Except in the transitional season, all underground open intermediary space in Chongqing needs to be fitted with heating, ventilating, and air conditioning (HVAC) devices to maintain thermal comfort. Therefore, thermal comfort was evaluated in terms of thermal discomfort degree and thermal discomfort time instead of predicted mean vote and predicted percentage dissatisfied (PMV-PPD). For the thermal environment response data, S_2_ was used to indicate the mean value of thermal discomfort time and thermal discomfort degree. The smaller the S_2_ value, the better the thermal environment.
3. Acoustic environment evaluation. It is generally accepted that the optimal reverberation time for sound emitters with an acoustic frequency of 500–2,000 Hz is 0.8–1.1 s (36). For the acoustic environment response data, S_3_ was used to indicate the average reverberation time of sound emitters with an acoustic frequency of 500–2,000 Hz. The larger the S_3_ value, the better the acoustic environment.
4. Physical environment evaluation. In this study, overall physical environment comfort was evaluated using the linear weighted sum method. The weighting process includes two stages. The first stage is to unify the order of magnitude of the acoustic, luminous, and thermal environment data, to avoid the results being biased toward higher mean values of environmental simulation. In the absence of the unification process, the S_2_ value is 22,870.73, and the S_1_ value is only 410.14. Direct analysis of overall physical environment comfort will make thermal environment response data “overwhelm” the luminous and acoustic environments. Therefore, the first stage consists of value minimization. Minimization means that the simulation result of each condition in the physical environment is divided by the minimum value (Equation 1) to unify the order of magnitude while the data trend is ensured. Snn denotes the response value after Sn is minimized.

(1) $$\begin{array}{l} S_{nn}=\frac{S_{n}}{minS_{n}} \end{array}$$

The second stage comprises weighting. At present, many scholars have studied differences in the influence of indoor heat, light, and sound environments on human comfort in different regions, buildings, and seasons. They found that the scores of importance varied greatly and no obvious law of importance of physical environment was evident (37, 38). In China, the new TOD complexes must meet the compliance requirements for green buildings. Therefore, the score of the physical environment (determined as per the Assessment Standard for Green Buildings, a Chinese national standard) was used as the weight of the linear weighted sum method (39); specifically, the importance scores of thermal, luminous, and acoustic environments were 25, 12, and 18, respectively, with a ratio of 2.083:1:1.5, respectively. The higher the average scores, the better the acoustic and luminous environments; the lower the average score, the better the thermal environment. S_4_ denotes the physical environment response value after Sn is calculated using the linear weighted sum method (Equation 2). The higher the S_4_ value, the better the environmental effect.


(2) $$\begin{array}{l} S_{4}=\left(S_{11}\times 1\right)-\left(S_{22}\times 2.083\right)+\left(S_{33}\times 1.5\right) \end{array}$$

## 3. Creating standard models

Standard models are a prerequisite for studying the physical environment of a building. A standard model is created in three stages. The first stage is the sampling stage, in which the target buildings are classified in detail using the function-oriented method, and the geometric dimensions. In addition, the buildings' spatial forms are measured, to increase the applicability and universality of the standard model. The second stage is the model building stage, in which the “skeleton” of the standard model is built by describing the characteristics of different types of buildings briefly through architectural modeling software (SketchUp was used in this study). The third stage is the boundary condition setting stage, in which the boundary conditions with spatial characteristics are entered into the simulation software to facilitate model simulation and optimization. Ecotect is considered a classical simulation tool, which is widely recognized for its accuracy and practicality. Specific parameters (e.g., timetables, area attributes, and material properties) are important boundary conditions for model simulations.

### 3.1. Field research

In this study, we preliminarily investigated 28 typical TOD complexes in Chongqing, and identified 11 target TOD complexes that fall within the scope of this study. Using a high-accuracy laser rangefinder, we measured some of their important parameters (e.g., floor height and spatial dimensions). Based on the spatial function orientation and measurement data of four types of buildings, the underground open intermediary space of TOD complexes in Chongqing was classified into four categories (Table 5) with the following characteristics:

1. TUAI. When the same bases are shared by different types of functional space (e.g., metro stations, intercity rail station buildings, and bus stops) and the main space of TOD complexes, underground open intermediary space is mainly presented in the form of underground atrium space. Such intermediary space is usually referred to as a “core of traffic,” which is characterized by great spatial height, many underground floors, many traversed floors, high clear height, and a large flow of people.
2. TCAI. When metro stations are planarly superposed with the bases of the main space of commercial buildings, underground open intermediary space is presented in the form of underground atrium space. Such space is characterized by people's short stay, complex streamline organization, and great spatial height. Underground atrium intermediary space improves space connectivity maximally, but weakens the functional diversity of intermediary space and sacrifices the flexibility of spatial arrangement.
3. TCSI. When metro stations are separated from the main buildings of TOD complexes due to the existing built environment, the form of intermediate space depends on the distance between them. When the distance between them is long or passageways exist in the space, underground passageways are the only choice. When metro stations are relatively near and suitable site conditions are available, open sunken plaza intermediary space can better connect the metro stations to the main buildings of TOD complexes. Sunken plaza intermediary space helps people to travel in a destination-oriented manner, namely, to reach the desired destinations directly without entering the interior of the main buildings, thus creating clear routes for people's flow.
4. THAI. When metro stations intersect with buildings' main space (e.g., high-speed rail station buildings or terminal buildings), underground open intermediary space is usually presented in the form of underground atrium space. Compared with TUAI and TCAI, the functions of THAI are more oriented toward vertical transportation beyond the waiting boundaries. Therefore, underground open intermediary space is not directly arranged inside metro station buildings, but distributed around the whole hub plazas. Such space is characterized by a few aboveground floors with low clear heights, and a few underground floors with high clear height.

**Table 5:** Position of four types of underground open intermediary space in TOD complexes.

**Number**	**Type**	**Profile view**	**Planar view**	**Representative building**
1	TUAI	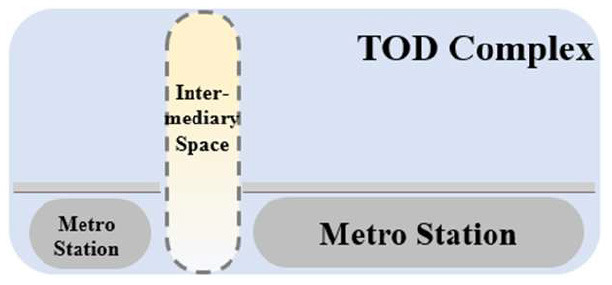	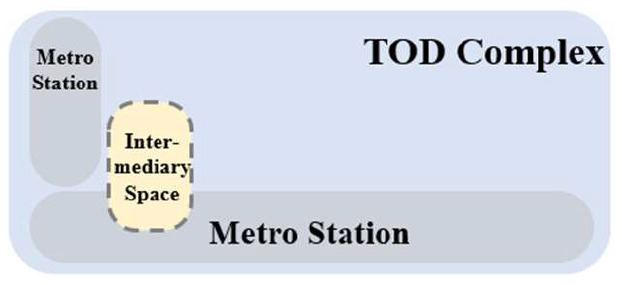	Shapingba station
2	TCAI	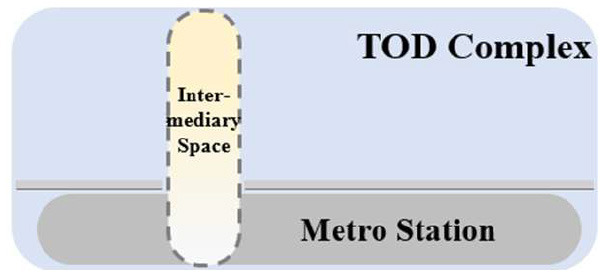	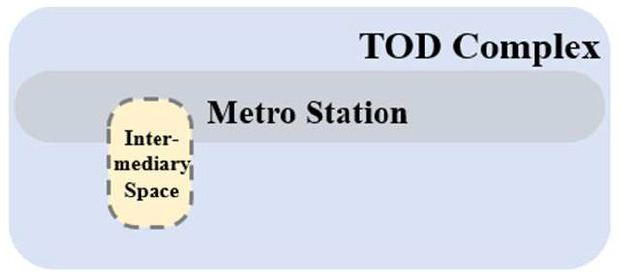	Xiaoshizi station
3	TCSI	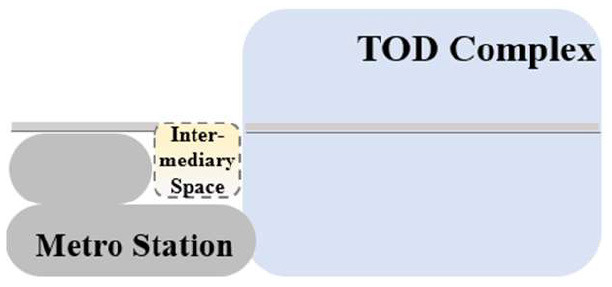	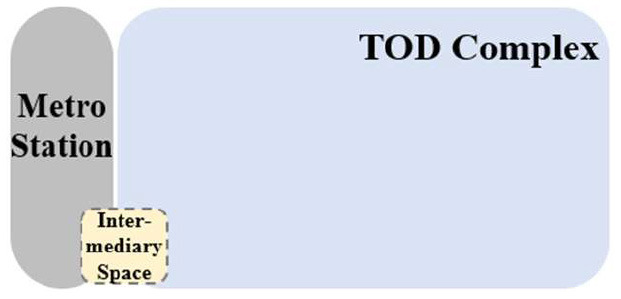	Shiqiaopu station
4	THAI	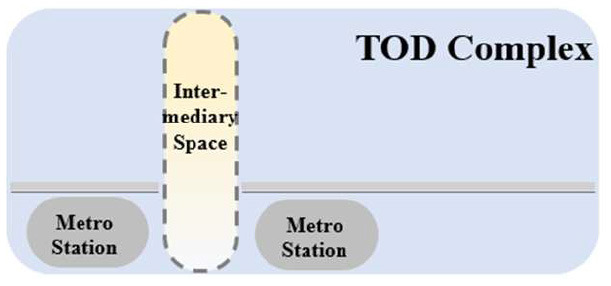	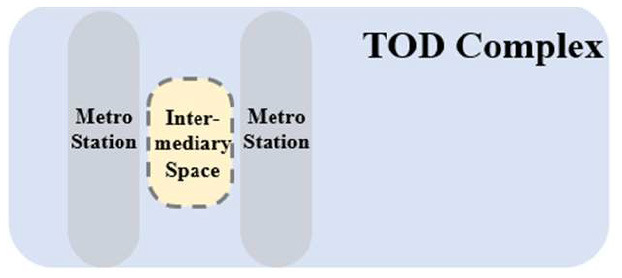	Chongqing West Railway Station

### 3.2. Standard model

The standard modeling approach is suitable for studies that obtain universal conclusions about future trends, and does not require high accuracy of experimental tools. Hence, we adopted this approach to meet its nature and needs (40, 41). Moreover, we constructed a standard model for the four types of underground open intermediary space in TOD complexes as mentioned in Section 3.1.

**Figure 2:**
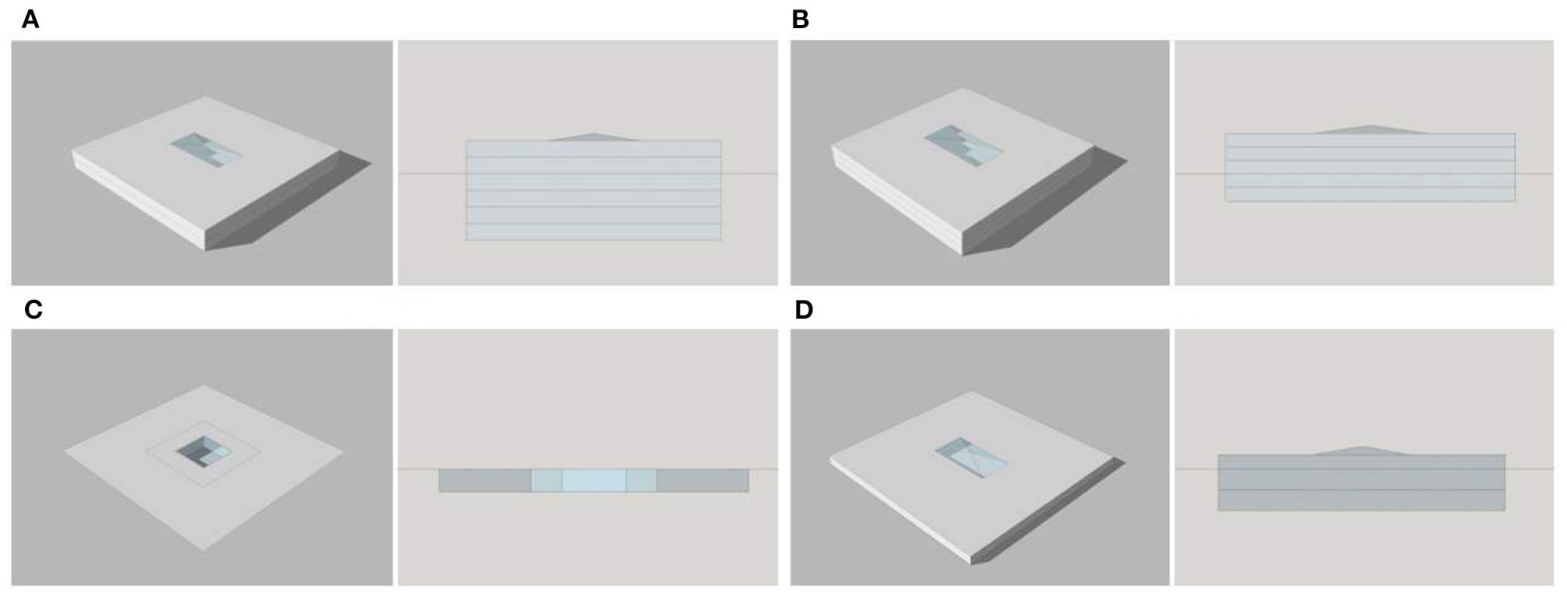
Aerial view and profile view of four standard models. Image source: self-drawn by the author. **(A)** TUAI. **(B)** TCAI. **(C)** TCSI. **(D)** THAI.

TUAI is densely developed, traverses many floors, and is lighted through side and top lighting (top lighting is adopted in most Chinese TUAIs) with few aboveground floors, but many underground floors. Therefore, the TUAI simulated by a standard model comprises two aboveground floors and four underground floors, has a floor height of 7 m and an atrium area of 800 m^2^, and covers a total area of 10,000 m^2^ (Figure 2A). TCAI has more aboveground floors but less floor height than TUAI. The survey data show that TCAI is characterized by an uneven distribution of the atrium area. As exemplified by the Shiyou Road Station, the skylights are linearly arranged and each skylight is small because the TCAI is long and narrow. Therefore, the TCAI simulated by a standard model comprises two underground floors and three aboveground floors, has an atrium area of 1,000 m^2^, and encompasses a total area of 10,000 m^2^ (Figure 2B).

TCSI is in direct contact with the external environment, so its luminous and thermal environments are susceptible to meteorological conditions. Hence, the geometric parameters of sunken plazas were optimized in this study. Due to their unique nature, the sunken plaza height and number of sunken floors are limited. The TCSI simulated by a standard model comprises one sunken floor, has a sunken plaza height of 7.5 m and sunken area of 431 m^2^, and encompasses a total area of 10,000 m^2^, with consistency between hole area and plaza area (Figure 2C). TOD hub complexes are limited in Chongqing. Hence, a standard model for THAI was constructed in this study, as exemplified by the Chongqing West Railway Station and Chongqing North Railway Station. Compared with TCAI, THAI has a greater floor height, fewer aboveground floors (because it is not directly connected with high-speed rail station buildings), and smaller volumes (usually one or two stories). Therefore, the THAI simulated by a standard model comprises two underground floors (floor height: 7.5 m) and one aboveground floor (floor height: 4 m), has a total height of 18 m and atrium area of 700 m^2^, and encompasses a total area of 10,000 m^2^ (Figure 2D).

### 3.3. Basic settlement in Ecotect

**Figure 3:**
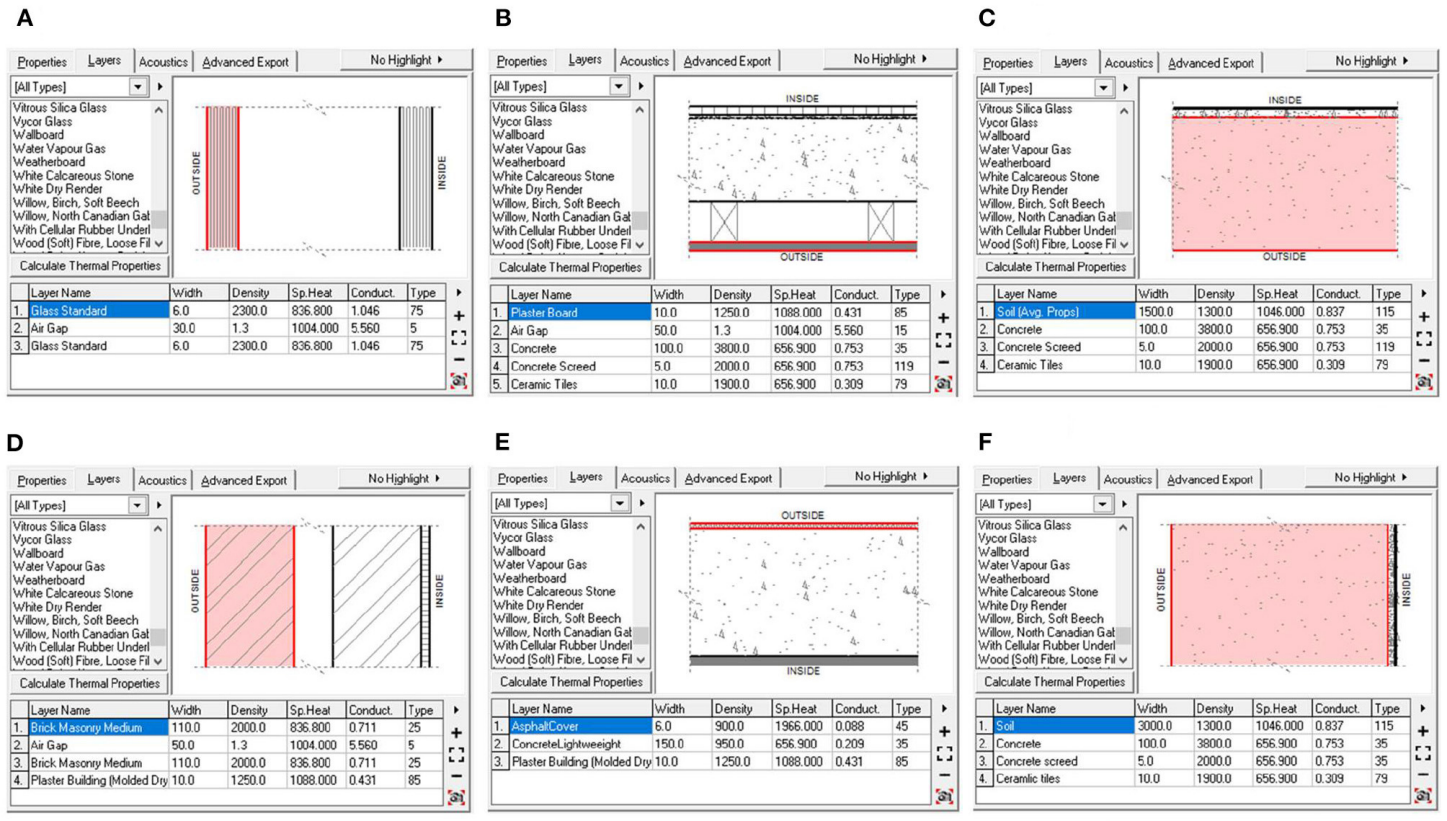
Profile view of the material composition layer. Image source: self-drawn by the author. **(A)** DoubleGlazed_LowE_AlumFrame. **(B)** ConcFlr_Tiles_Suspended. **(C)** ConcSlab_Tiles_OnGround. **(D)** DoubleBrickCavityBlaster. **(E)** ConcreteRoof_Asphalt. **(F)** Underground wall.

Ecotect is widely used in the simulation of indoor environments and has been confirmed as sufficiently accurate in reflecting trends in an indoor environment, within reasonable boundary conditions (42, 43). Timetables, area attributes, and material properties are important boundary conditions for numerical simulation, and are the basis for calculating buildings' usage characteristics and operation times. This study focused on optimizing space comfort by changing the design parameters, while removing the boundary condition disturbances of the active system. The basic boundary conditions of the modes are divided into three parts:

1. Timetable setting. According to Chongqing's rail transit service schedule (44), a year was divided into standard workdays and standard weekend days. The starting time of space use was set to 6:00 and its ending time of usage was set to 22:30, according to the operating time of TOD complexes.
2. Material property setting. Material properties are an important influencing factor for the physical environment of space, and the material price is an important indicator of the effectiveness of materials. Experimental conditions must remain consistent during the simulation experiment to prevent the fluctuation of simulation results arising from the material price. Figure 3 presents the settings of different materials.
3. Parameter difference description. TCSI differs from TUAI, TCAI, and THAI in spatial morphology, but is basically consistent with them in terms of functionality, peak flow, timetable, and material definition. The differences in parameter settings are mainly manifested in the following aspects:

4. Differences in material property setting. TUAI, TCAI, and THAI usually have aboveground sections, so the simulation of their underground sections differs from that of TSCI. We first selected the material in Figure 3F for the outdoor area, and set the aboveground wall separately from the underground wall.
5. Differences in aboveground wall modeling. The aboveground part of TUAI, TCAI, and THAI is usually far away from the boundaries of buildings, and their light is blocked by the surrounding functional areas (e.g., shops and restaurants). Therefore, we did not set side windows.
6. Differences in simulated area scope. For TUAI, TCAI, and THAI, the simulated areas of the thermal and acoustic environments were changed. Due to the solidness and closure of space, the simulated areas for thermal and acoustic environments were established in areas 10 m beyond underground space rather than specifying large or small areas.

## 4. Results

**Figure 4:**
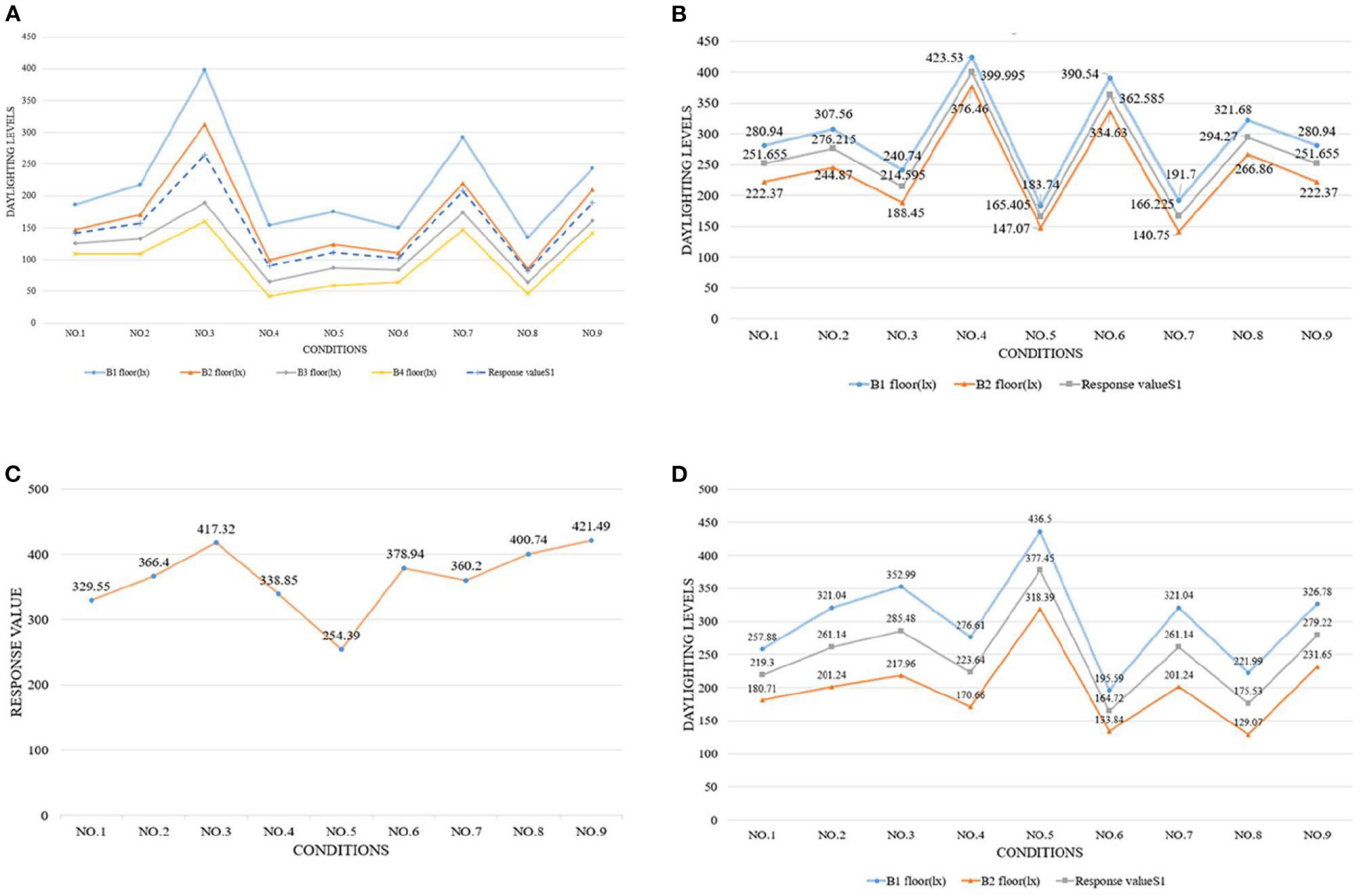
Luminous environment simulation results and response values for **(A)** TUAI, **(B)** TCAI, **(C)** TCSI, and **(D)** THAI. Image source: self-drawn by the author.

**Figure 5:**
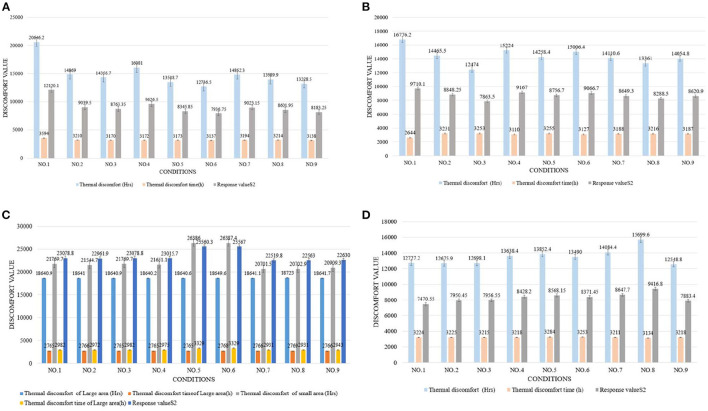
Thermal environment simulation results and response values for **(A)** TUAI, **(B)** TCAI, **(C)** TCSI, and **(D)** THAI. Image source: self-drawn by the author.

**Figure 6:**
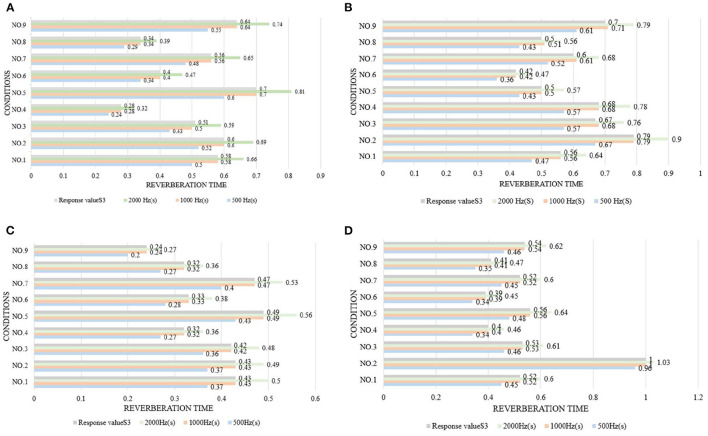
Acoustic environment simulation results and response values for **(A)** TUAI, **(B)** TCAI, **(C)** TCSI, and **(D)** THAI. Image source: self-drawn by the author.

Figures 4–6 show the simulation results of the luminous, thermal, and acoustic environments obtained by the four models, including average natural illuminance, thermal discomfort degree, thermal discomfort time, and reverberation time under nine conditions. Using the evaluation method mentioned in Section 2.3, we analyzed the simulation results. Accordingly, we determined the environment response values (S_1_-S_4_), priorities of impact factors for the luminous, thermal, acoustic, and physical environments, and the best and worst condition combinations.

### 4.1. TUAI

The impact factors of different floors can be prioritized by comparing the simulation results of the average natural illuminance of the belowground level 1 (B1) to belowground level 4 (B4) floors (Figure 4A). The study results show that the luminous environment is significantly influenced by the W/A ratio, and the average natural illuminance of each flow is positively correlated with the W/A ratio. This is in line with the expectations. The weight of the level factor of the profile inclination angle varies with the increase in the floor number (Figure 8A).

**Figure 7:**
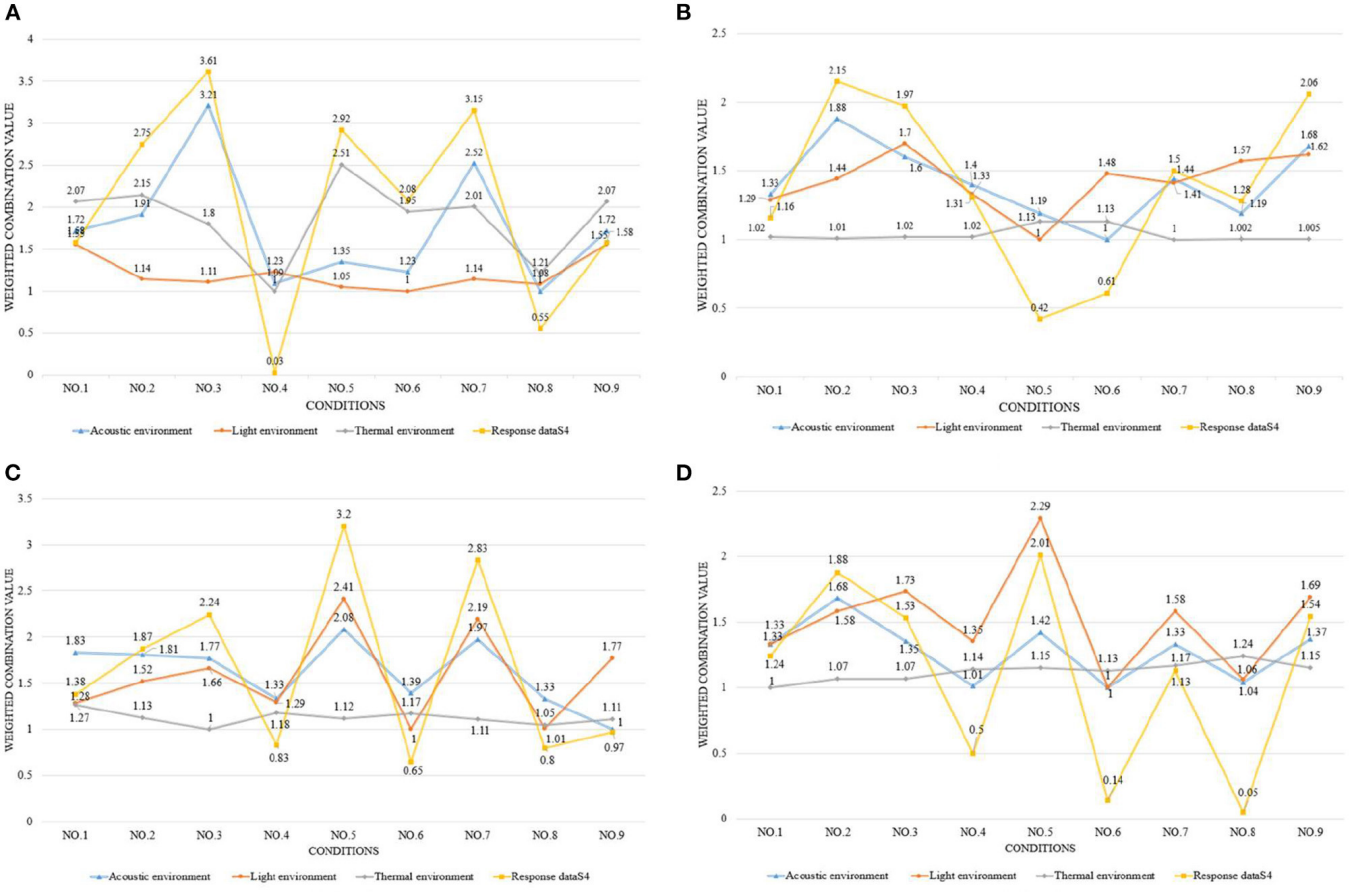
Physical environment simulation results and response values for **(A)** TUAI, **(B)** TCAI, **(C)** TCSI, and **(D)** THAI. Image source: self-drawn by the author.

**Figure 8:**
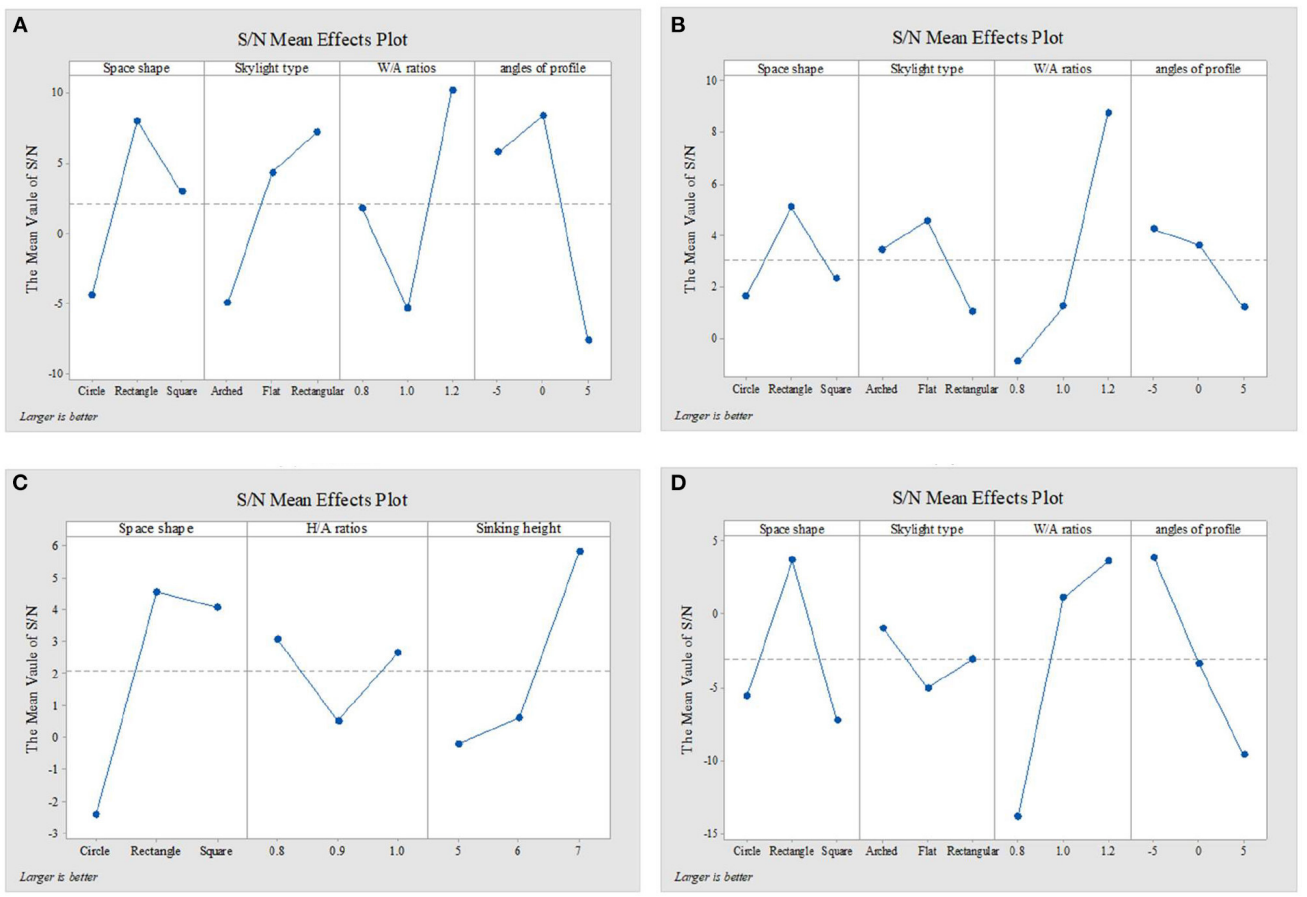
Physical environment response values for **(A)** TUAI, **(B)** TCAI, **(C)** TCSI, and **(D)** THAI. Image source: self-drawn by the author.

**Figure 9:**
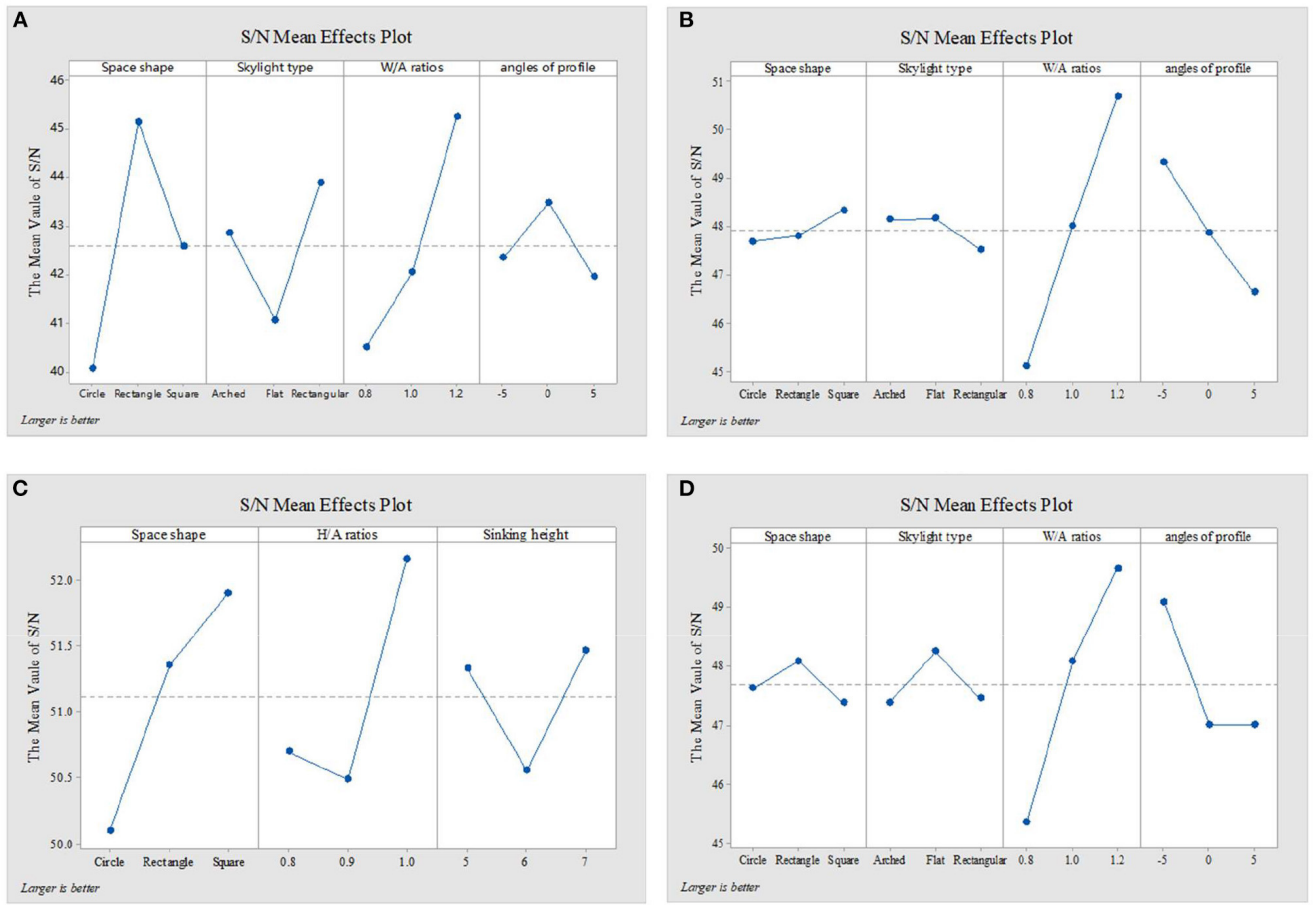
Luminous environment response values for **(A)** TUAI, **(B)** TCAI, **(C)** TCSI, and **(D)** THAI. Image source: self-drawn by the author.

**Figure 10:**
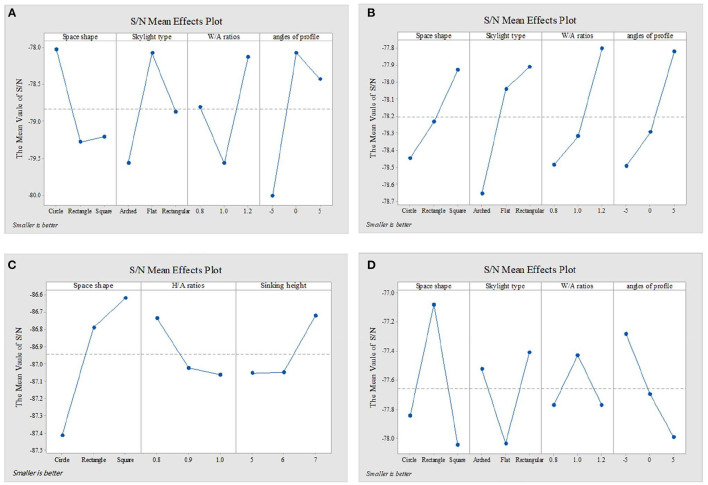
Thermal environment response values for **(A)** TUAI, **(B)** TCAI, **(C)** TCSI, and **(D)** THAI. Image source: self-drawn by the author.

**Figure 11:**
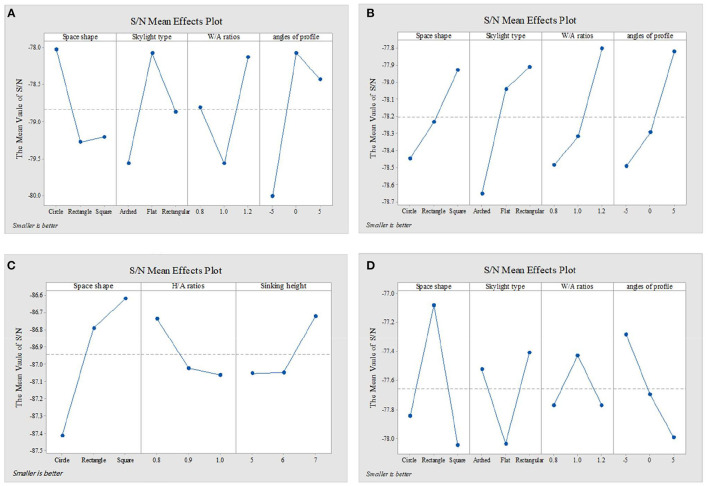
Acoustic environment response values for **(A)** TUAI, **(B)** TCAI, **(C)** TCSI, and **(D)** THAI. Image source: self-drawn by the author.

The analysis of the differences between floors can help designers understand the mechanism of how the impact factors affect the luminous environment, but the wholeness of the luminous environment should not be ignored. Objectively, the importance of B1–B4 floors is the same. Therefore, the response value S_1_ of the overall luminous environment indicates the mean value of the average natural illuminance of the four underground floors. As shown in Figure 9A, the priorities for impact factors are affected when the luminous environment is optimized. According to the analysis results, the best condition combination for luminous environment optimization is Condition 10 and the worst condition is Condition 11. The thermal environment was simulated for floors B1–B4, but the simulation results were not analyzed separately for different floors (Figure 10). To optimize the thermal environment of TUAI, the best condition combination is Condition 12, and the worst condition combination is Condition 1. Owing to the differences in the reverberation times calculated based on different sound frequencies, the average reverberation time calculated under three sound frequencies (500, 1,000, and 2,000 Hz) was used as the acoustic environment response value (the three sets of data covered the middle and high-frequency bands generated by musical instruments and human voices). Figure 6 shows that the reverberation time is all lower than the range of comfortable reverberation time (i.e., 0.8–1.1 s). Therefore, it is necessary to prolong the reverberation time moderately through spatial design. Figure 11A shows the variation trend of the level factors of the impact factors for acoustic environment optimization. To optimize the acoustic environment of TUAI, the best condition combination is Condition 13, and the worst condition combination is Condition 4. Figure 8A shows the variation trend of the level factors of the impact factors for physical environment optimization. Evidently, it is highly correlated with that of the acoustic environment (Figure 7A). To optimize the TUAI's physical environment, the best condition combination is Condition 10, and the worst condition combination is Condition 4.

### 4.2. TCAI

According to the variation trend of average natural illuminance in the B1 and B2 floors (Figure 4B), we can conclude that the level factors of impact factors in the B1 and B2 floors produce the same influence on the luminous environment. This indicates that at the spatial depth of ~10 m, the priority of impact factors and variation trend of their level factors are not influenced by the underground depth. Figure 9B shows the variation trend of the level factors of the impact factors for luminous environment optimization. To optimize the luminous environment of TUAI, the best condition combination is Condition 10, and the worst condition combination is Condition 11. Figure 10B shows the variation trend of the level factors of the impact factors for thermal environment optimization. To optimize the thermal environment of TCAI, the best condition combination is Condition 12, and the worst condition combination is Condition 13. To optimize the acoustic environment of TCAI (Figure 11B), the best condition combination is Condition 14, and the worst condition combination is Condition 15. To optimize the physical environment of TCAI (Figure 7B), the best condition combination is Condition 16, and the worst condition combination is Condition 17.

### 4.3. TCSI

The model for TCSI involves only one underground floor, so the luminous environment response value (S_1_) of TCSI denotes the average natural illuminance within a 750-m^2^ plane (Figure 4C). To optimize the luminous environment of TCSI (Figure 9C), the best condition combination is Condition 9, and the worst condition combination is Condition 5. As mentioned in Section 3.3, comfort optimization for the thermal environment of TCSI was simulated on a large-area and small-area basis. The results of large-area thermal environment evaluation reflect the overall thermal environment impact of underground open intermediary space, while the results of small-area thermal environment evaluation directly reflect the change in the thermal environment of underground open intermediate space arising from thermal radiation. The analysis results (Figure 5C) show that the level factors of the impact factors for the thermal environment of TCSI are relatively similar, implying a limited potential for thermal environment optimization. To optimize the thermal environment of TCSI, the best condition combination is Condition 10, and the worst condition combination is Condition 6. The acoustic environment simulation for TCSI is different from that for the three other types of space. In this study, the acoustic environment of TCSI was simulated on a small-area basis, for two reasons. First, the results of large-area simulation for simplified space tend to be consistent, failing to provide effective results. Second, the utility of TCSI is more significant than that of large-area space, as manifested by the fact that appropriate reverberation time is required to ensure high acoustic quality for frequent commercial activities held in small-area spaces. For the acoustic environment optimization of TCSI (Figure 6C), space shape affects the acoustic environment of TCSI most significantly, because none of the mean values of impact factors fall within the range of 0.8–1.1 s. To optimize the acoustic environment of TCSI (Figure 11C), the best combination of conditions is Condition 11, and the worst condition combination is Condition 6. Figure 8C shows the integrated analysis results of luminous, thermal, and acoustic environment optimization of TCSI; the best condition combination is Condition 12, and the worst condition combination is Condition 13. The physical environment response value (S_4_) of TCSI shows that the variation trend of the level factors of various impact factors is consistent with that of the acoustic environment (Figure 7C). Therefore, the best and worst condition combinations for the physical environment are the same as those for the acoustic environment.

### 4.4. THAI

A comparison of the luminous environment simulation results of TUAI, TCAI, and TCSI (Figure 9) indicates that the ratio of window to courtyard always significantly impacts the luminous environment. The level factors of skylight types (e.g., rectangular or arched) installed in floors B1 and B2 were consistent. However, rectangular skylights facilitated a luminous environment more steadily than arched skylights on the B2 floor. The variation trend of the level factors of space, shape, and profile inclination angle clearly differed between the B1 and B2 floors (Figure 8D). When the overall luminous environment is analyzed, the luminous environment response value (S_1_) denotes the mean value of the average natural illuminance of the B1 and B2 floors. To optimize the luminous environment of THAI (Figure 9D), the best condition combination is Condition 10, and the worst condition combination is Condition 11. To optimize the thermal environment of THAI, the best condition combination is Condition 12, and the worst condition combination is Condition 8. To optimize the acoustic environment of THAI (Figure 11D), the best condition combination is Condition 2, and the worst condition combination is Condition 13. To optimize the physical environment of THAI (Figure 7D), the best condition combination is Condition 10, and the worst condition combination is Condition 8.

## 5. Discussion

The results of condition analysis show that TUAI, TCAI, TCSI, and THAI are affected by impact factors to varying degrees. For comfort optimization for the luminous, thermal, acoustic, and physical environments in the four types of space, we calculated the numerical differences between the best and worst condition combinations, analyzed the potential for comfort optimization in different environments in the four types of space, and proposed appropriate optimization strategies.

### 5.1. Optimization strategy

#### 5.1.1. TUAI

Figure 9A shows that the Delta values of four impact factors (i.e., space shape, skylight type, W/A ratio, and profile inclination angle; the same below; for the luminous environment) in TUAI are in the ratio of 3.32:2.39:3.10:1, and space shape is a primary factor for optimizing the luminous environment. Owing to the differences in spatial depth between the B1 and B4 floors, we comparatively analyzed the signal-noise ratio of the luminous environment in the B1–B4 floors, finding that the influence of the level factor of profile inclination angle on the luminous environment of the B2 floor was ~0. Both the weights of impact factors and priorities of their level factors vary from floor to floor. In the B3 and B4 floors, space shape outweighs the W/A ratio to become a primary factor. The weight of the W/A ratio is negatively correlated with spatial depth; in the B3 floor, its downtrend is alleviated, and the priorities of impact factors tend to be stable. Except for the W/A ratio, the influence of other impact factors on the luminous environment tends to decline from the B3 floor. Hence, to optimize the luminous environment of TUAI, designers must first determine the space shape and W/A ratio. The most effective solution is to adjust the profile inclination angle at a high underground depth.

Figure 10A shows that the Delta values of four impact factors (for the thermal environment) in TUAI are in the ratio of 1.88:2.81:1:1.1, and skylight type and space shape are primary factors for optimizing the thermal environment. Therefore, arched skylights and a rectangular shape should not be used for optimizing the thermal environment. Comfort optimization for the thermal environment can reduce the annual thermal discomfort degree by 32% and shorten the annual thermal discomfort time by 250 h; hence, the thermal environment of TUAI has great optimization potential. The higher the W/A ratio, the lower the annual thermal discomfort degree; this is due to the fact that the thermal discomfort degree reduced by solar radiation in winter exceeds the thermal discomfort degree produced by solar radiation in summer. This conclusion is true under certain circumstances. The thermal environment response value comprises thermal discomfort degree and thermal discomfort time, and the response value to thermal discomfort degree is far larger than that of thermal discomfort time. Therefore, the analysis of the thermal environmental response data cannot fully represent the change in thermal discomfort time; rather, the priorities of impact factors for thermal discomfort time are different from those for thermal discomfort degree.

Figure 11A shows that the Delta values of four impact factors (for the acoustic environment) in TUAI are in the ratio of 1:1.34:1.25:3.4, and profile inclination angle is a primary factor for optimizing the acoustic environment. The profile inclination angle of −5° can expand spatial volume, which is consistent with the principle for the calculation of reverberation time. The comparison between Condition 13 (the best condition combination) and Condition 4 (the worst condition combination) shows that the highly-targeted optimization of the acoustic environment can increase the reverberation time by 248%, but the actual reverberation time is still far from the target range of 0.8–1.1 s. What's worse, the luminous environment under Condition 13 is poor; specifically, the natural illuminance of TUAI under Condition 13 is reduced by 16.7% compared with that under Condition 4. Thus, in practice, it is not advisable to give priority to acoustic environment optimization.

Figure 8A shows that the Delta values of the four impact factors (for the physical environment) in TUAI are in the ratio of 1.02:1:1.23:1.32, and the physical environment is basically correlated with the four impact factors to the same degree. During the design process of TUAI, the profile inclination angle of 0° and W/A ratio of 1.2 are advised, and the four impact factors can all be changed according to other conditions if conflicts with other factors arise. As shown in Figures 9–11, the primary impact factors for the acoustic, luminous, and thermal environments are different, fundamentally explaining why the weights of the four impact factors for the physical environment are basically the same. The acoustic environment is influenced by the acoustic absorptivity of the building materials, so it can be modified by changing the materials in practice. Hence, the regulating mechanism for the luminous and thermal environments has received much attention. In TUAI, the thermal and luminous environments are unified in terms of profile inclination, angle, and W/A ratio. For the thermal environment, thermal radiation can be enhanced in winter through an appropriate W/A ratio and profile inclination angle; for the luminous environment, the daylighting area can be expanded by changing its values. For the luminous and acoustic environments, the trend of the level factors of the four impact factors is basically consistent without contradictory factors. For the acoustic and thermal environments, the trend of the level factors of space shape is contrary, whereas the trend of the level factors of skylight type and W/A ratio is the same.

After the physical environment of TUAI is optimized, the luminous environment response value (S_1_) under the best condition combination is 3.4 times greater than that of the worst condition combination, and the acoustic environment response value (S_3_) under the best condition combination is 2 times greater than that of the worst condition combination. The optimization potential for the thermal environment is lower than that of the luminous and acoustic environments, and the thermal environment response value (S_2_) is almost the same between the best and worst condition combinations. However, the thermal discomfort time of the best condition combination is longer than that of the worst condition combination, implying that the physical environment of TUAI shortens annual thermal discomfort time, but consumes more resources in summer. Hence, designers should first optimize the luminous environment of TUAI rather than its thermal environment.

#### 5.1.2. TCAI

Figure 9B shows that the Delta values of four impact factors (for the luminous environment) in TCAI are in the ratio of 1.02:1:8.84:4.29, and the W/A ratio is a primary factor for optimizing the luminous environment. With an increase in spatial depth, the variation trend of the Delta values of the four impact factors in TCAI is the same as that in TUAI. However, the weights of the impact factors in TCAI are significantly different from those in TUAI because of the differences in the total number of belowground floors and number of aboveground floors. The simulation results under Condition 10 (the best condition combination) and Condition 11 (the worst condition combination) show that the acoustic, luminous, and thermal environments under the best condition combination for the luminous environment are all better than those under the worst condition combination for the luminous environment. The luminous environment response value (S_1_) under the best condition combination is 2.64 times that under the worst condition combination. That being said, the luminous environment response value (average illuminance) under the worst condition combination still meets the requirements for daylighting Class IV specified in the Standard for Daylighting Design of Buildings. Therefore, the luminous environment of TCAI does not need to be specially optimized if TCAI has no functional areas such as reading areas, exhibition halls, entry halls, and waiting halls.

The four impact factors for optimizing the thermal environment of TCAI are almost equally important, and their Delta values are in the ratio of 1:1.42:1.31:1.29 (Figure 10B). Comfort optimization for the thermal environment can reduce the annual thermal discomfort degree by 21% and shorten the annual thermal discomfort time by 120 h. Like TUAI, the trend of thermal discomfort time is not completely consistent with that of the thermal environmental response values (S_3_).

Figure 11B shows that the Delta values of four impact factors (for the acoustic environment) in TCAI are in the ratio of 1.78:1.59:2.44:1. Compared with TUAI, space, shape, and W/A ratio affect the acoustic environment of TCAI more significantly due to there being fewer traversed floors and lower spatial height. Studies show that reverberation time is affected more significantly by the profile inclination angle in spaces with high floor heights and high overall heights, which implies larger spatial volumes. Therefore, the profile inclination angle affects the acoustic environment less significantly in the two underground floors with a floor height of 5 m.

Figure 8B shows that the Delta values of the four impact factors (for the physical environment) in TCAI are in the ratios of 1.15:1.16:3.18:1, and the physical environment is strongly correlated with the W/A ratio, but weakly correlated with the three other impact factors. To optimize TCAI, the W/A ratio of 1.2 must first be achieved, while other impact factors can be adjusted according to other conditions. The W/A ratio is a primary influencing factor for luminous and acoustic environments, and a second influencing factor for the thermal environment; the W/A ratio of 1.2 is the best level factor. Therefore, a high W/A ratio is beneficial to improving the physical environment of the underground section of TCAI, and should be prioritized. In the luminous and thermal environments, the level factors of the W/A ratio and space shape trend are consistent. However, the trend of level factors of the profile inclination angle is contrary to each other. The profile inclination angle is a primary contradictory factor for the luminous and thermal environments, and skylight type is a secondary contradictory factor for luminous and thermal environments. When energy consumption is not considered, the profile inclination angle of 0° can balance the luminous and thermal environments, and is also the best level factor for the acoustic environment; hence, it should be prioritized. The contradictory factor between the acoustic, luminous, and thermal environments is space shape; specifically, a rectangular shape can prolong reverberation time. After TUAI's physical environment is optimized, the luminous environment response value (S_1_) under the best condition combination is 2.5 times greater than that of the worst condition combination, and the acoustic environment response value (S_2_) under the best condition combination is 1.7 times greater than the worst condition combination, but the thermal environment comfort degree is improved only by 3%. This conclusion is similar to that of TUAI.

#### 5.1.3. TCSI

Figure 9C shows that the Delta values of three impact factors (i.e., space, shape, H/A ratio, and sunken plaza height; the same below; for the luminous environment) in TCSI are in the ratio of 2.22:2.07:1. To optimize TCSI's luminous environment, space shape and W/A ratio should be prioritized, and the additional average illuminance caused by the limited change in sunken plaza height should be ignored. The comparison of simulation results under Condition 9 (the best condition combination) and Condition 5 (the worst condition combination) shows that comfort optimization for the luminous environment also improves the comfort of the thermal and acoustic environments. Generally, the trend lines of the H/A ratio and sunken plaza height are positively correlated with the luminous environment response value (S_1_), but the two impact factors show clear inflection points in Figure 9C. The H/A ratio tends to decline slowly in the value section of 0.8–0.9, indicating that sectional change affects luminous environment comfort slightly. When TCSI meets the basic illuminance requirements, the H/A ratio of 0.8 is preferred for reducing the annual thermal discomfort degree. However, luminous environment comfort is improved significantly when the H/A ratio is in the value section of 0.9–1.0. Therefore, the H/A ratio of 1.0 is preferred when the illuminance requirement is high or the average illuminance fails to reach the expected value. In the trend line of sunken plaza height, the inflection point is at the level factor of 6 m, with a downtrend first and then an uptrend.

Studies show that at the sunken plaza height of 6–7 m, the improvement in luminous environment comfort is mainly due to internal reflection, and the improvement in luminous environment comfort from internal reflection offsets the loss of luminous environment comfort arising from a change in sunken plaza height. When TCSI has a high requirement for its luminous environment and the fencings outside the sunken plazas are not made of high-reflectivity materials (e.g., glass curtain walls), a condition combination with a low spatial depth should be prioritized to improve the luminous environment comfort, and avoid the factor combination of circular shape plus a H/A ratio of 0.9 plus a sunken plaza height of 6 m.

Figure 10C shows that the Delta values of the three impact factors (for the thermal environment) in TCSI are in the ratio of 2.42:1:1.02, and space shape is a primary factor for optimizing the thermal environment. To optimize thermal environment comfort, space shape must first be determined. The simulation results under Condition 10 (the best condition combination) and Condition 6 (the worst condition combination) show that thermal environment optimization also improves acoustic environment comfort slightly. The luminous environment response value (S1) under Condition 10 is smaller than that of Condition 6, but the average natural illuminance under Condition 10 exceeds 150 lx (Figure 4C). After thermal environment comfort is optimized, average natural illuminance is reduced, but is nearly 120 lx higher than that of Condition 5 (254.39 lx). The thermal environment response value involves four groups of data (including small-area thermal discomfort degree and small-area thermal discomfort time); in large areas, actual projects are uncertain and active equipment is inevitable. The comparison of the mean value of the signal-noise ratio between the four groups of data shows that the overall trend of the thermal environment is the same as the trend of the thermal environment response value in small areas, but is significantly different from that in large areas. The analysis results show that in large areas, the level factors of space shape are prioritized in the descending order of rectangular shape, circular shape, and square shape, and the level factor of H/A ratio are prioritized in the descending order of 0.8, 1, and 0.9, and the level factor of sunken plaza height are prioritized in the descending order of 6, 7, and 5 m. Based on the plot of signal-noise ratio and differences in the thermal environment response value, this study concluded that the comfort optimization design for the thermal environment can ignore the thermal environment fluctuations under the H/A ratio of 0.8–0.9 and sunken plaza height of 5–6 m. Additionally, comfort optimization for the thermal environment can reduce the annual thermal discomfort degree by 14% at the cost of 12% average natural illuminance.

Figure 11C shows that the Delta values of three impact factors (for the acoustic environment) in TCSI are in the ratio of 8.38:1:13.84, and sunken plaza height and space shape are primary factors for optimizing the acoustic environment. Sound emitters are arranged on two diagonal lines, so circular shape has the advantages such as short acoustic reflection distance and quick reach to sound-absorbing materials, thus taking less time for the sound decay of 60 Db (45). Likewise, the increased sunken plaza height leads to an increase in spatial volume and transmission path, thus prolonging the reverberation time. The comparison between the best and worst condition combinations for the acoustic environment shows that the acoustic environment of TCSI has a high optimization potential, and the optimized reverberation time increases by 200%, very close to the comfortable reverberation time range. However, acoustic environment optimization leads to a decline in the luminous environment response value; this phenomenon is consistent with the results of acoustic environment optimization for TUAI. The comfort optimization design for the acoustic environment should give priority to sunken plaza height, followed by space shape. Although the H/A ratio affects the reverberation time slightly, the delay of 16.7 ms nevertheless falls within the perceptible range of 12–35 ms. Therefore, it is not advisable to completely ignore the influence of the H/A ratio on the acoustic environment of TCSI.

Figure 11C shows that the Delta values of three impact factors (i.e., space shape, H/A ratio, and sunken plaza height; for the physical environment) in TCSI are in the ratio of 2.79:1:2.37, and the physical environment is strongly correlated with space shape and sunken plaza height. The level factors of the H/A ratio for the thermal environment are prioritized in the descending order of 0.8, 0.9, and 1.0; this is completely contrary to the priorities of level factors of the H/A ratio for the luminous environment. Considering the year-round thermal environment, a smaller H/A ratio is not always better; from a year-round perspective, the luminous and thermal environments do not completely conflict. In the optimization design of TCSI, the H/A ratio should be considered in conjunction with other factors. For the optimization of TUAI, comfort optimization for the physical environment has a high potential, and can partly meet the related national standards for spatial luminous and acoustic environments, thus reducing the annual discomfort time significantly. The luminous environment response value (S_1_) under the best condition combination is 1.3 times that under the worst condition combination; the acoustic environment response value (S_2_) under the best condition combination is 1.6 times that under the worst condition combination. The thermal environment optimization for TCSI is significantly more effective than that for TUAI and TCAI, with a 21% improvement in thermal comfort and a reduction of nearly 350 h in thermal discomfort time in surrounding areas.

#### 5.1.4. THAI

Figure 9D shows that the Delta values of four impact factors (i.e., space shape, skylight type, W/A ratio, and profile inclination angle; the same below; for the luminous environment) in THAI are in the ratio of 1:1.23:6.07:2.98, and the W/A ratio is a primary factor for optimizing the luminous environment. Therefore, for projects with high requirements for luminous environment comfort, designers should first determine the W/A ratio, and may ignore the influence of space shape and skylight type on the luminous environment depending on other conditions. The overall illuminance of the B2 floor is lower than that of the B1 floor, but the optimization efficiency of different impact factors is basically the same between the two floors (for example, the optimization efficiency of profile inclination angle is 64% in the B1 floor and 66% in the B2 floor). The comparison of simulation results between Condition 10 (the best condition combination) and Condition 11 (the worst condition combination) shows that the best condition combination for luminous environment optimization is also superior to the worst condition combination in terms of acoustic environment optimization, with an optimization efficiency of 98% for luminous environment comfort and 55% for acoustic environment comfort. Like TCAI, average natural illuminance under the worst condition combination for luminous environment optimization also meets the requirements for the top daylight of Class IV specified in the Standard for Daylighting Design of Buildings, and highly-targeted luminous environment optimization can be ignored unless otherwise necessitated.

Figure 10D shows that the Delta values of four impact factors (for the thermal environment) in THAI are in the ratio of 2.85:1.85:1:2.09, and space shape is a primary factor for optimizing the thermal environment. Therefore, comfort optimization for the thermal environment of THAI should give priority to profile inclination angle and space shape, and give up the optimal W/A ratio if necessary. The comparison of simulation results between Condition 12 (the best condition combination) and Condition 8 (the worst condition combination) for the thermal environment shows that the best condition combination can reduce energy consumption by 18% at most without consideration of the efficiency of active equipment, but increase the annual thermal discomfort time by 74 h.

Figure 11D shows that the Delta values of four impact factors (for the acoustic environment) in THAI are in the ratio of 2.26:1:1.87:1.81, and space shape is a primary factor for optimizing the acoustic environment. The comparison of simulation results between Condition 2 (the best condition combination) and Condition 13 (the worst condition combination) shows that the acoustic environment response value (S_3_) under the best condition combination is 169% larger than that under the worst condition combination, and both the luminous and thermal environment comfort under the best condition combination is higher than that under the worst condition combination. Evidently, acoustic environment optimization for THAI does not directly contradict with its luminous or thermal environment.

Figure 8D shows that the Delta values of four impact factors (for the physical environment) in THAI are in the ratio of 9.94:1:14.18:10.73, and the physical environment is strongly correlated with skylight type, but weakly correlated with the three other impact factors. The acoustic, luminous and thermal environments are weakly correlated with skylight type, but strongly correlated with the three other factors at the same frequency, explaining the great differences in the Delta values of the four impact factors for the physical environment of THAI. Therefore, the optimization design for THAI may ignore the influence of skylight type on the indoor physical environment. In the luminous and thermal environments, skylight type shows opposite trends in Figures 9D, 10D. However, we can conclude that there are no contradictory factors for the luminous and thermal environments because skylight type is weakly correlated with them. For the luminous and acoustic environments, the level factors of the W/A ratio, profile inclination angle, and skylight type show a consistent trend and do not contradict with each other. For the acoustic and thermal environments, the level factors of profile inclination angle show a consistent trend, and skylight type is a contradictory factor. In terms of the H/A ratio, the thermal environment of THAI is different from that of TUAI and TCAI, indicating that the luminous-thermal contradiction is more obvious in the shallow underground space. After the physical environment of TUAI is optimized, the luminous environment response value (S_1_) under the best condition combination is 2.1 times that under the worst condition combination, and the acoustic environment response value (S_3_) under the best condition combination is 1.54 times that under the worst condition combination. Like TCSI, the thermal environment comfort of TUAI has a high optimization potential, and is improved by 18% through appropriate optimization.

### 5.2. Comparison of the four models

In this study, TUAI is characterized by high underground depth with many underground floors, high aboveground height with few aboveground floors, and high clear height; TCAI is characterized by low underground depth with few underground floors, high aboveground height with many aboveground floors, and low clear height; THAI is characterized by low underground depth with few underground floors, low aboveground height with few aboveground floors, and great clear height. Additionally, space shape, W/A ratios, and H/A ratios are classified as planar geometric elements, profile inclination angle and sunken plaza height are classified as solid geometric elements, and other impact factors are classified as detail construction elements. For the physical environment of TUAI, the impact factors are prioritized in the descending order of solid geometric elements, planar geometric elements and detail construction elements. For the physical environment of TCAI, the impact factors are prioritized in the descending order of planar geometric elements, detail construction elements and solid geometric elements. For the physical environment of TCSI, the impact factors are prioritized in the descending order of solid geometric elements and planar geometric elements. For the physical environment of THAI, the impact factors are prioritized in the descending order of planar geometric elements, solid geometric elements, and detail construction elements.

The comparison of simulation results for TUAI, TCAI, and THAI reveal the following:

1. For luminous environment, underground open intermediary space with many underground floors is more susceptible to solid geometric elements; underground open intermediary space with low underground depth and few underground floors is more susceptible to planar geometric elements.
2. For thermal environment, underground open intermediary space with high aboveground height is more susceptible to detail construction elements; underground open intermediary space with low aboveground depth is more susceptible to planar geometric elements; underground open intermediary space with few aboveground and underground floors is more susceptible to the external environment, and its optimization efficiency is low.
3. For acoustic environment, underground open intermediary space with high traversed height and high clear height (i.e., high underground depth and high aboveground height) is more susceptible to solid geometric elements in terms of reverberation time; underground open intermediary space with medium traversed height and low clear height or with low traversed height and high clear height is more susceptible to planar geometric elements.
4. For physical environment, underground open intermediary space with high traversed height is more susceptible to solid geometric elements, and underground open intermediary space with low traversed height is more susceptible to planar geometric elements.
5. The luminous environment optimization efficiency of TUAI is far higher than that of TCAI, TCSI, and THAI. The thermal environment optimization efficiency of TCSI and THAI is lower than that of TUAI and TCAI. However, TCSI has no specific spatial boundaries and THAI is characterized by high spatial height and large area; hence, thermal environment optimization is less necessary for TCSI and THAI than for TUAI and TCAI. The acoustic environment optimization efficiency of TCAI is the second highest. However, its floor height is the lowest among the four types of space, so its optimized acoustic environment is nevertheless far from the comfortable range. Therefore, the necessity of acoustic environment optimization is directly proportional to floor height.

### 5.3. Design implication

The results of this study show that the underground open intermediary space in TOD complexes requires a balance between the luminous, thermal, and acoustic environments. In practice, optimizing the underground open intermediate space in TOD complexes can bring more natural light to their underground part, reduce the annual thermal discomfort time, and improve the quality of public broadcasting systems. Active systems can create a more comfortable physical environment, but improving passive performance through reasonable building parameter design is the core approach to the balance between the physical environment of TOD complexes and their construction cost (46, 47).

From the perspective of use, underground open intermediary space in TOD complexes is also an important spatial node (48). Usually, the underground part of TOD complexes is very large, and underground open intermediary space in TOD complexes is an important “locator” for people (49), helping them determine their relative positions quickly. Underground open intermediary space in TOD complexes is also a core component of a three-dimensional traffic system, which helps people find their way or evacuate and reach their destinations quickly through escalators and stairs. Moreover, it also provides the possibility of diverse ground-floor forms, weakens the building boundaries, and integrates TOD complexes with the external environment fully. This study proposes practical principles for the optimization design of TUAI, TCAI, TCSI, and THAI. The comparative analysis of simulation results between them helps designers understand the variation pattern of the priorities of impact factors under other conditions (e.g., underground floor number and underground depth) and thus design underground open intermediate spaces in TOD complexes more efficiently. The findings of this study will not only help provide a healthier and more comfortable physical environment for people in TOD complexes, but also promote the development and popularization of the TOD model.

## 6. Conclusion

Based on a survey of 28 TOD complexes in Chongqing, we found that an underground open intermediary space can effectively improve the physical environment comfort of TOD complexes. According to the survey results, we determined the variation range of impact factors and their level factors, and constructed four space models (including TUAI, TCAI, TCSI, and THAI models). Taking the comfort optimization of the physical environment as the primary objective and comfort optimization of the luminous, thermal, and acoustic environments as the auxiliary objective, we used numerical simulation to identify the best influencing factor combinations under various circumstances, and sought appropriate design strategies for comfort optimization. The findings of this study are summarized as follows:

1. Space shape is a primary influencing factor for luminous and thermal environments. Except in the TUAI model, the priorities of level factors of space shape are consistent in the comfort optimization of the luminous and thermal environments. Therefore, a circular shape should be avoided in the architectural design.
2. The H/A ratio and W/A ratio are contradictory factors between the luminous and thermal environments, and annual discomfort time and thermal discomfort degree in summer are negatively correlated with the priorities of the level factors of the impact factors for the luminous and thermal environments.
3. Profile inclination angle and sunken plaza height are primary impact factors for the acoustic environment. In space models with a floor height of more than 7 m, the two impact factors can improve acoustic environment comfort far more significantly than other impact factors.
4. For comfort optimization of the physical environment, skylight type is an influencing factor with a low weight.
5. In the four space models, the optimization efficiency of luminous and acoustic environments is 200%. However, the comparison of simulation results and comfort range shows that only the comfort optimization of the luminous environment of TUAI is highly necessary, whereas the comfort optimization of the acoustic environment of TCAI is less necessary. Therefore, the physical environment comfort of TUAI can be optimized based on the conclusions in Section 5.1; the thermal environment comfort of TCAI needs to be optimized, and its luminous and acoustic environment comfort can be optimized through active equipment and change of materials; the luminous environment comfort of TCSI and THAI does not need to be optimized.

The significance of this study is limited for several reasons (e.g., limited surveyed regions and number of surveys, undiversified climatic conditions, and exclusion of active equipment). TOD complexes are necessarily fitted with large HVAC systems. In this study, the highly-targeted comfort optimization of the thermal environment ignored the heat loss in winter. Hence, the comfort optimization measures of the thermal environment may change in practice. Ecotect is a common architectural physics simulation tool, and its simulation results may be overestimated, but the overall trend is consistent. Future studies may adopt the dynamic Taguchi method and introduce climatic conditions and noise factors of active equipment to comprehensively discuss how to improve the habitability and comfort of the indoor physical environment of TOD complexes.

## Data availability statement

The original contributions presented in the study are included in the article/Supplementary material, further inquiries can be directed to the corresponding authors.

## Author contributions

DL: writing—original draft, investigation, and supervision. HY: investigation, simulation, and data analysis. CX: polish, proofreading, and picture production. CN: project administration, writing—review and editing, and data analysis. LT: writing—review and editing. All authors contributed to the article and approved the submitted version.
