# Supplementary material for: Optimization of underground open intermediary space comfort in TOD complexes: A case study of Chongqing, China
Source: Front Public Health. 2023 Feb 24;11:1108750. doi: 10.3389/fpubh.2023.1108750 (PMC10003338; doi:10.3389/fpubh.2023.1108750)
Supplement: Supplementary file 1 [file Table_1.docx]

Appendix A. The indoor environment response data of TUAI

| Conditions | S_1_ | S_2_ | S_3_ | S_4_ |
| --- | --- | --- | --- | --- |
| 1 | 142.21 | 11401.30 | 580.0 | 1.59 |
| 2 | 157.47 | 8397.50 | 603.3 | 2.75 |
| 3 | 264.92 | 8129.35 | 506.7 | 3.62 |
| 4 | 90.45 | 8992.10 | 280.0 | 0.04 |
| 5 | 111.36 | 7711.25 | 703.3 | 2.92 |
| 6 | 102.04 | 7309.35 | 546.7 | 2.09 |
| 7 | 208.17 | 8384.35 | 563.3 | 3.16 |
| 6 | 82.33 | 7959.15 | 340.0 | 0.55 |
| 9 | 189.26 | 7555.65 | 643.3 | 3.59 |
| 10 | 307.15 | 6437.45 | 570.0 | 4.47 |
| 11 | 71.10 | 6652.14 | 673.3 | 2.33 |
| 12 | 153.63 | 7984.60 | 563.3 | 2.61 |
| 13 | 75.32 | 8583.65 | 693.3 | 2.18 |

Appendix B. The indoor environment response data of TCAI

| Conditions | S_1_ | S_2_ | S_3_ | S_4_ |
| --- | --- | --- | --- | --- |
| 1 | 212.98 | 9181.30 | 433.3 | 1.38 |
| 2 | 251.66 | 8202.05 | 430.0 | 1.88 |
| 3 | 276.22 | 7212.90 | 420.0 | 2.25 |
| 4 | 214.60 | 8545.00 | 316.7 | 0.84 |
| 5 | 399.99 | 8105.70 | 494.3 | 3.21 |
| 6 | 165.41 | 8441.30 | 330.0 | 0.65 |
| 7 | 362.59 | 8011.70 | 466.7 | 2.84 |
| 6 | 166.23 | 7645.30 | 316.7 | 0.80 |
| 9 | 294.27 | 7983.50 | 236.7 | 0.97 |
| 10 | 396.62 | 7122.45 | 516.7 | 4.29 |
| 11 | 150.22 | 11785.05 | 320.0 | 1.79 |
| 12 | 357.63 | 6584.25 | 460.0 | 4.23 |
| 13 | 216.36 | 8127.00 | 393.3 | 1.45 |
| 14 | 340.78 | 7631.25 | 356.7 | 2.83 |
| 15 | 213.30 | 7592.20 | 246.7 | 1.29 |
| 16 | 390.70 | 7458.40 | 526.7 | 3.55 |
| 17 | 166.37 | 7655.35 | 310.0 | 1.76 |

Appendix C. The indoor environment response data of TCSI

| Conditions | S_1_ | S_2_ | S_3_ | S_4_ |
| --- | --- | --- | --- | --- |
| 1 | 329.55 | 21929.40 | 556.7 | 1.16 |
| 2 | 366.40 | 21714.25 | 786.7 | 2.16 |
| 3 | 417.32 | 21929.40 | 670.0 | 1.92 |
| 4 | 338.85 | 21867.65 | 676.7 | 1.64 |
| 5 | 254.39 | 24341.50 | 500.0 | 0.43 |
| 6 | 378.94 | 24347.60 | 416.7 | 0.62 |
| 7 | 360.20 | 21380.40 | 603.3 | 1.50 |
| 6 | 400.74 | 21422.95 | 500.0 | 1.29 |
| 9 | 421.49 | 21488.20 | 703.3 | 2.10 |
| 10 | 370.32 | 21429.40 | 486.6 | 1.12 |
| 11 | 352.13 | 21952.30 | 780.0 | 2.05 |
| 12 | 348.12 | 21882.25 | 676.7 | 1.67 |
| 13 | 267.70 | 24244.65 | 416.7 | 0.19 |

Appendix D. The indoor environment response data of THAI

| Conditions | S_1_ | S_2_ | S_3_ | S_4_ |
| --- | --- | --- | --- | --- |
| 1 | 219.30 | 6825.75 | 523.3 | 1.24 |
| 2 | 261.14 | 7305.45 | 663.0 | 1.89 |
| 3 | 285.48 | 7313.55 | 533.3 | 1.54 |
| 4 | 223.64 | 7784.60 | 400.0 | 0.51 |
| 5 | 377.45 | 7911.35 | 560.0 | 2.01 |
| 6 | 164.72 | 7720.85 | 393.3 | 0.14 |
| 7 | 261.14 | 8005.50 | 523.3 | 1.14 |
| 6 | 175.53 | 8790.00 | 410.0 | 0.05 |
| 9 | 279.22 | 7239.80 | 540.0 | 1.55 |
| 10 | 360.00 | 7453.55 | 630.0 | 3.31 |
| 11 | 181.70 | 7103.70 | 406.7 | 1.49 |
| 12 | 214.38 | 7388.70 | 550.0 | 2.14 |
| 13 | 196.62 | 7840.35 | 393.3 | 1.30 |
